# Supplementary figures and images for: CAF-associated genes putatively representing distinct prognosis by in silico landscape of stromal components of colon cancer
Source: PLoS One. 2024 Apr 1;19(4):e0299827. doi: 10.1371/journal.pone.0299827 (PMC10984474; doi:10.1371/journal.pone.0299827)

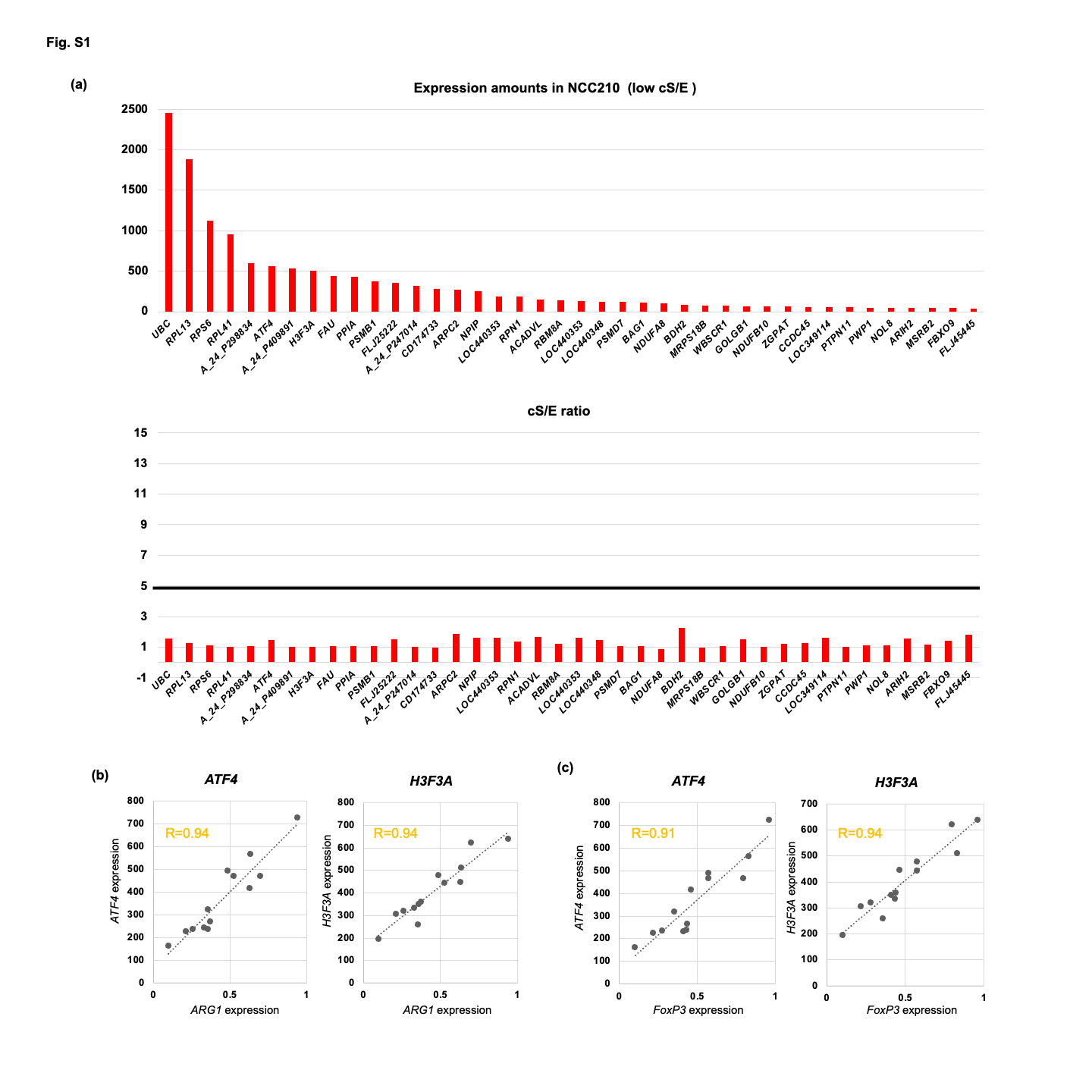

Supplement: S1 Fig — (TIF) [file pone.0299827.s001.tif]
