# Supplementary material for: CAF-associated genes putatively representing distinct prognosis by in silico landscape of stromal components of colon cancer
Source: PLoS One. 2024 Apr 1;19(4):e0299827. doi: 10.1371/journal.pone.0299827 (PMC10984474; doi:10.1371/journal.pone.0299827)
Supplement: Attachment — Submitted filename: Review for PO.docx [file pone.0299827.s014.docx]

Review for “CAF-associated genes putatively representing distinct prognosis by in silico landscape of stromal components of colon cancer.”

Summary

This study conducted an *in silico* analysis of stromal components in primary colon cancer, focusing on markers of cancer-associated fibroblasts and other tumor-associated elements. Authors found that CAF-associated genes generally have a negative impact on prognosis and overlap partially with genes associated with tumor-associated endothelia and myeloid cells, but not with cytotoxic T lymphocytes genes. The research highlights the prognostic significance of various stromal elements in the tumor microenvironment of colon cancer, offering new insights into its clinical features.

Comments

1. The statement "Cancer is a genetic disease" is inappropriate, being partially correct, but it requires some clarification. Cancer is indeed caused by genetic mutations, but these mutations can be either inherited (germline mutations) or acquired (somatic mutations).
2. Objectives or aims of in silico analysis of CRC tumors were not clearly stated, what’s lacking is the rational between the experiment and the identification of CAF-associated genes.
3. The resolution of data is not high enough to allow reviewers to acquire the information and make further evaluations. For example, the texts in all Figures are not readable.
4. For the presenting of the microarray-based database, the authors are encouraged to describe the big picture or broader view prior to listing specific examples. Other data or examples may also need to be discussed briefly.
5. The authors are suggested to provide more interpretation or explanations of their results and discuss the biological impact of their findings rather than simply displaying the number of genes being identified from their experiments.
6. Many genes have been identified and mentioned in this manuscript, however more detailed information regarding the known function of the top hits is lacking. To increase the biological significance of current study, the authors can briefly hypothesize potential involvement of top genes in CRC tumor pathogenesis or therapeutic applications.
