# Supplementary material for: CAF-associated genes putatively representing distinct prognosis by in silico landscape of stromal components of colon cancer
Source: PLoS One. 2024 Apr 1;19(4):e0299827. doi: 10.1371/journal.pone.0299827 (PMC10984474; doi:10.1371/journal.pone.0299827)
Supplement: Attachment — Submitted filename: Response to Reviewers.docx [file pone.0299827.s015.docx]

PONE-D-23-42477　　CAF-associated genes putatively representing distinct prognosis by in silico landscape of stromal components of colon cancer.

PLOS ONE

Thank you for your positive comments. I responded to your advice one by one, and revised our manuscript extensively for publication.

Reviewer #1: The manuscript "CAF-associated genes putatively representing distinct prognosis by in silico landscape of stromal components of colon cancer" provides an in-depth analysis of the tumor microenvironment (TME) in colon cancer. It emphasizes the prognostic relevance of cancer-associated fibroblasts (CAF) and their associated genes, exploring their interactions with other components like tumor-associated endothelia, myeloid cells, and T lymphocytes. The study utilizes in silico analysis of micro-dissected stromal gene signatures, offering novel insights into the clinical features of the TME in colon cancer. This approach could significantly enhance the understanding of colon cancer progression and prognosis, potentially guiding more effective therapeutic strategies.

However, the paper should consider and discuss some additional details. Minor revisions based on the following points are recommended for acceptance:

Thank you for your positive comments.

**1. How were the cancer-associated fibroblast genes (CAFGs) selected, and could the selection criteria bias the results?**

[Response]

Thank you for your comment. CAFGs were selected as (1) stroma/epithelia ratio=10 or beyond in GSE35602 (microdissected CRC tumors), (2) R-index with *SPARC* in cancer stroma=0.9 or beyond, which were included in the original paper on page 4, line 105-109 in the revised paper. As we prioritized expression amounts, we compared the expression values of NCC210, which has the highest gene expression of the 13 cases of GSE35602. 76 genes with expression levels of 100 or beyond were further used for prognostic analysis, and 62 genes were proved to be poor prognostic factor. Such information was also included in the original paper on page 4, line 115-122. The selection of CAFG was based on *SPARC* among the CAFG in this study, however the results may be biased if other CAFGs were used. Therefore, such limitation was added on page 15-16, line 498-502 in the revised paper.

**2. How do different components of the TME, such as tumor-associated endothelia and myeloid cells, interact with CAFGs, and what implications does this have for colon cancer progression?**
[Response]

Thank you for your comment. Ben figures identified overlap between CAFG and other components of the TME as shown in Fig. 1b. Such genes may be considered to represent CAFG affecting angiogenic function like *ANXA1* (CAF and TAE). In our data, their prognostic relevance was confirmed, suggesting that overlapped features of CAF and TAE represent more aggressiveness than TAE markers themselves in CRC. We have added this interpretation on page 13, line 418-420.

**3. The manuscript mentions the subclassification of CAFGs based on fibrosis. How does this subclassification enhance the understanding of colon cancer prognosis?**

[Response]

Thank you for your comment. Fibrosis representing COL family genes were enriched in CAFG with the highest AUC, so colon cancer with the highest COL family gene expression showed the most aggressive phenotypes, suggesting COL family gene is involved to assist cancer cells rather than protection from cancer cells. Previous reports have also supported our current data. We described such hypothesis on page 14, line 432-434.

**4. Given the study's findings, what are the potential clinical applications, particularly in terms of targeted therapies or prognostic markers?**

[Response]

Thank you for your comment. Our findings clarified that both CAFG and CTLG were the potent prognostic factors, while other components of the TME were dependent on either factor. Among them, CTLG may be a good marker to predict Immune checkpoint Inhibitor efficacy, while CAFG remains elusive to control. Among CAFG, the COL family is a particularly poor prognostic factor, suggesting that they have excellent potential as prognostic biomarkers. We added this description on page 15, line 481-486.

Reviewer #2: This study conducted an in silico analysis of stromal components in primary colon cancer, focusing on markers of cancer-associated fibroblasts and other tumor-associated elements. Authors found that CAF-associated genes generally have a negative impact on prognosis and overlap partially with genes associated with tumor-associated endothelia and myeloid cells, but not with cytotoxic T lymphocytes genes. The research highlights the prognostic significance of various stromal elements in the tumor microenvironment of colon cancer, offering new insights into its clinical features.

Thank you for your pertinent comments.

Comments

**1. The statement "Cancer is a genetic disease" is inappropriate, being partially correct, but it requires some clarification. Cancer is indeed caused by genetic mutations, but these mutations can be either inherited (germline mutations) or acquired (somatic mutations).**

[Response]

Thank you for your pertinent comment. Cancer is a genetic disease, which was either inherited (germline mutations) or acquired (somatic mutations). We added this description on page 2, line 48-53.

**2. Objectives or aims of in silico analysis of CRC tumors were not clearly stated, what’s lacking is the rational between the experiment and the identification of CAF-associated genes.**

[Response]

Thank you for your comment. Objectives of in silico analysis of CRC tumors were to obtain comprehensive results from the same assessment, and public database can be accessed and obtained by other researchers. We included these objectives on page 3, line 69-70.

**3. The resolution of data is not high enough to allow reviewers to acquire the information and make further evaluations. For example, the texts in all Figures are not readable.**

[Response]

Thank you for your advice. We will immediately improve the quality/resolution of all the figures. Also, the resolution is poor in the integrated PDF file, but the resolution seems to be clear in the individual TIF files. We would appreciate your confirmation.

**4. For the presenting of the microarray-based database, the authors are encouraged to describe the big picture or broader view prior to listing specific examples. Other data or examples may also need to be discussed briefly.**
[Response]

Thank you for your suggestion. As you pointed out, some parts are difficult to comprehend, so we have added representative examples of each TME-associated genes as broader view on page 4-5, line 124-132, instead of only listing them.

**5. The authors are suggested to provide more interpretation or explanations of their results and discuss the biological impact of their findings rather than simply displaying the number of genes being identified from their experiments.**

[Response]

Thank you for your thoughtful feedback. We acknowledge the importance of providing a more in-depth interpretation of our results and discussing the biological implications of our findings. In response to your comments, we would like to add descriptions on page 15, lines 492-497, regarding the biological impact of our findings.

**6. Many genes have been identified and mentioned in this manuscript, however more detailed information regarding the known function of the top hits is lacking. To increase the biological significance of current study, the authors can briefly hypothesize potential involvement of top genes in CRC tumor pathogenesis or therapeutic applications.**

[Response]

Thank you for your comment. Some of the CAFG listed in Fig. 1a were discussed in the manuscript (*PLAT* and *ANXA1*: page13, line 415-420. *COL5A2*, *COL5A1*, *COL12A1*, *COL3A1* and *COL1A1*: page14, line 434-436. *SPARC* and *TAGLIN*: page14, line 437-439). Additionally, we have provided details about certain genes (*COL1A1* and *RAB31*) and their impacts on CRC, along with references and discussion on page 15, lines 487-491.
